# Intestinal parasitic infections and their potential risk factors among prison inmates in Valencia, Spain

**DOI:** 10.1186/s12879-023-08613-1

**Published:** 2023-09-19

**Authors:** Carla Muñoz-Antoli, María José Irisarri-Gutiérrez, Lucrecia Acosta, María José Bonet, J. Guillermo Esteban, Rafael Toledo

**Affiliations:** 1 Department of Pharmacy and Pharmaceutical Technology and Parasitology, Faculty of Pharmacy, University of Valencia, Valencia, Spain; 2 Department of Preventive Medicine, Public Health and Microbiology, Faculty of Medicine, Autonomous University of Madrid, Madrid, Spain; 3 Parasitology Area, Department of Agrochemistry and Environment, Miguel Hernández de Elche University, Alicante, Spain; 4 Medical Deputy Director, Picassent Penitentiary Center, Valencia, Spain

**Keywords:** Intestinal parasites, Prison inmates, *Blastocystis* sp., Risk factors, Spain

## Abstract

**Background:**

Despite the fact that prison inmates are a population at higher risk than other groups of suffering from intestinal parasite infections in relation to their living conditions, information about these diseases in prison environments is still scarce. Herein, we analyze the status of intestinal parasite infections in a Spanish prison.

**Methods:**

A cross-sectional study involving 528 inmates was conducted from April to June 2022 among inmates at Centro Penitenciario Picassent (Valencia, Spain). Stool specimens were examined using the direct wet mount technique, the formol-ether concentration technique, and the Ziehl-Neelsen staining method. We used STATA 16.1 for data analysis. We consider a p-value less than 0.05 significant at a 95% confidence level.

**Results:**

Of the 528 inmates (471 men and 57 women; a mean age of 41.94 years) enrolled in the study, 83 (15.7%) were infected. Only six species of protozoa were detected. The gut potential microeukaryotic pathobiont *Blastocystis* sp. was the predominant parasite, accounting for 37 (44.6%) of the infections. Gut parasite amebas (6.6%) and pathobionts (5.3%) were more prevalent than flagellates (2.3%). The prevalence of infection with pathogenic species (8.9%) was similar to that of non-pathogenic species (8.7%). Infection among men (15.2%) was higher than in women (0.6%) (p < 0.0001). In multivariate analysis, the country of birth (AOR = 0.31, 95% CI = 0.18–0.52) and the time spent in prison (AOR = 1.83, 95% CI = 1.06–3.14) were statistically significant associated to intestinal parasite infections (p < 0.0001 and p = 0.028, respectively).

**Conclusion:**

This study found low levels of intestinal parasite infections in the CPP which could be indicative of the implementation of sanitary measures in prison environments in Spain. The less time spent in prison favor the risk of having infection while the Spanish nationality of inmates could reduce the risk of infection. The main recommendation would be to introduce routine parasitological tests upon foreigners entering prison.

## Background

Intestinal parasite infections are among the most neglected tropical diseases, causing over 3.5 billion infections and significant morbidity in about 450 million people [1]. The majority of these infections occur in vulnerable groups in association with limited healthcare, poor nutrition, poverty, a low level of community and personal hygiene, and limited access to safe water or sanitary facilities [2–5]. These facts determine that intestinal parasite infections are highly prevalent in developing countries [6]. In high-income countries intestinal parasite infections have been commonly overlooked, making it difficult to estimate the real incidence of these diseases in the general population. Most of the surveys have focused on particularly vulnerable groups including children, the elderly, immigrant population, and immunosuppressed people [7–10].

Prison inmates constitutes a vulnerable group since they live under conditions that can facilitate transmission of intestinal parasites [11]. Prison inmates commonly represent marginalized communities that live in conditions such as overcrowding, inadequate sanitary conditions or, even high-risk behaviors that make them more susceptible to parasite infections than the general population [12].

Surprisingly, there is very little information about the status of intestinal parasite infections in prisons, especially in high-income countries. Most of the studies on this topic have been performed in developing countries from Africa, Asia or Latin America [1, 3, 5, 11, 13–24]. Only a few surveys were conducted in developed countries, in the last century in Canadian, Finish and Spanish prisons [25–27]. Despite significant efforts made in the last decades to improve the conditions in penal institutions in developed countries, this constitutes an important gap in our knowledge of the sanitary status of prisons.

Herein, we provide epidemiological data on the prevalence of intestinal parasites and associated factors among inmates of Centro Penitenciario Picassent (Valencia, Spain). Renewed and up-to-date information on the epidemiology of intestinal parasite infections in more vulnerable groups such as prisoners may significantly contribute towards improving the health condition of such an at-risk group.

## Materials and methods

### Study area and population

We conducted a cross-sectional study to determine the prevalence of intestinal parasites and associated risk factors among inmates of Centro Penitenciario de Picassent (CPP), Valencia (Spain), over 3-months period, from April to June 2022.

CPP (-0.458300 longitudinal and 39.358300 latitudinal coordinates) is one of the main prison of Spain housing about 2.100 inmates, coming to host up to 22 different nationalities of origin of the inmates. CPP is a transit center to other Spanish penitentiary centers in such a way that moments of great saturation have been denounced, in the 35 total modules available (a maximum of 1.330 cells are available). The CPP was chosen as the study site due to its accessibility from the University of Valencia (convenient for sample transportation) and high population of inmates. Moreover, the prison management is very supportive of measures to further improve the wellbeing of their inmates.

Our source population included all adult males and females prisoners, of various jail terms available during the study period, except those with security restrictions. Before starting the sampling, CPP medical authorities were contacted, and a request for cooperation was made after explaining the objective of the study.

### Sample size

As there are no preliminary results of the CPP, according to the estimated prevalence (p) of about 10% published in the Madrid reference study [27], with a sampling error (d) of 5% and a confidence level (z) of 1.96 (95% confidence interval), the sample size was calculated according to the formula:

n = N (z2pq/d2)/([N-n + z2pq/d2])

For the entire CPP inmate population (N = 2100 inmates), a statistically valid sample size would be at least 130 fecal samples or, what is the same, the participation of at least 130 inmates. Finally, a total of 528 inmates participated in the study, which represents 25% of the total prison population.

### Questionnaires and sample collection

All the 528 participating inmates delivered signed consents for voluntary participation. For the collection of sociodemographic (age, sex, country of birth, educational level, date of entry in prison, prison permit) and epidemiological (intestinal symptomatology, chronic diseases) data, a basic structured questionnaire was used, with simple and individual answers, which were delivered to the inmate population participating in the study, although help for its correct completion was offered to those who request it. All questionnaires had an alpha-numeric identification code, so that the total anonymity of the participants was maintained. The identities of the participants remained restricted within the prison environment. The results of the present study were submitted to the CPP medical authorities for further action. Treatment for positive cases was administered by the authorized doctors in the CPP.

During every collection visit, the researcher was accompanied by prison wardens who provided protection and assistance. Plastic stool containers were handed out to all 528 participants. The filled stool containers were collected over the next two days in refrigerated containers. Within 1–2 hours’ post-collection, the fresh faecal samples were transferred to the laboratory at the Department of Parasitology, Faculty of Pharmacy, Valencia (Spain). In the present study, only one stool specimen was collected from each participant. Once the faecal samples arrived at the laboratory, a small portion of each fresh faecal sample was transferred into a 2 ml microcentrifuge tube, and kept at -20 °C until a future molecular study, and the rest was half preserved with saline and the other half fixed with 10% formalin. Both the questionnaire and stool sample were linked by the alpha-numeric identification code.

### Laboratory procedures

528 preserved faecal samples were first, examined using the direct wet mount technique and, secondly, 2 gr faecal material were processed by in-house formalin ether concentration technique for the presence of intestinal protozoa and helminths [28]. Two drops of the sediment were observed on a glass slide, covered with a cover-slip and viewed (at 100× and 400× magnification) under light microscopy. The sediment from each sample was analyzed simultaneously by two different researchers. Modified Ziehl-Neelsen staining was performed for detection and confirmation of coccidians.

### Data analysis

Data analysis was done using STATA 16.1. The prevalence of intestinal parasite infections was calculated. Bivariate (Crude Odds Ratio = COR) and multivariate (Adjusted Odds ratio = AOR) analyss were run to determine the association and strength of association of variables with parasite infections, respectively. A significant value of p < 0.05 was used for all the tests. Variables with a p value of < 0.05 in bivariate analysis were used for the multivariable regression analysis model.

## Results

### Prevalence of intestinal parasites among inmates of CPP, Valencia, Spain

The prevalence of infection reached 15.7% (83/528) in the total population studied (Table 1). Only protozoan, with a spectrum of six species, were detected. The most predominant species in the entire population of prisoners studied was the gut microeukaryotic potential pathobiont *Blastocystis* sp. (7.0%) (37/528). Moreover, among the group of 83 infected inmates, *Blastocystis* sp. infection appears with a frequency of 44.6% (37/83) and *Giardia intestinalis* with one of 12% (10/83).

**Table 1:** Distribution of intestinal parasite infections among inmates of CPP, Valencia, Spain, 2022

	Infection rates of inmates	
	n/N	% (CI 95%)
Total infection	83/528	15.7 (12.8–19.0)
*Blastocystis*	37/528	7.0 (5.0-9.4)
*Endolimax nana*	30/528	5.7 (3.9–7.9)
*Entamoeba coli*	21/528	3.9 (2.5–5.9)
*Giardia intestinalis*	10/528	1.9 (0.9–3.4)
*Entamoeba hartmanni*	4/528	0.8 (0.2–1.8)
*Chilomastix mesnili*	3/528	0.6 (0.1–1.5)
*Blastocystis + E. nana*	6/528	1.1 (0.2–2.4)
*Blastocystis + E. coli*	3/528	0.5 (0.0-1.1)
*Blastocystis + E. nana + E. coli*	1/528	0.2 (0.0-0.9)
*Blastocystis + E. nana + E. hartmanni*	1/528	0.2 (0.0-0.9)
*G. intestinalis + E. nana*	1/528	0.2 (0.0-0.9)
Total Single infections	64/528	12.1 (9.5–15.1)
Total Mixed infections	19/528	3.6 (2.2–5.5)
Total with pathogens/pot. pathogenic species	47/528	8.9 (6.4–11.3)
Total with no pathogens	46/528	8.7 (6.3–11.1)
With both (pathogens/pot. pathogenic species and no pathogens)	10/528	1.9 (0.9–3.4)
Only with pathogens/pot. pathogenic species	37/528	7.0 (5.0-9.4)
Only with no pathogens	36/528	6.8 (4.9–9.2)
Only with amebas	35/528	6.6 (4.5–8.7)
Only with flagellates	12/528	2.3 (1.2–3.8)
Only with pathobiont	28/528	5.3 (3.6–7.5)
With Amebas + flagellates	1/528	0.2 (0.0-0.9)
With Amebas + pathobiont	9/528	1.7 (0.8–3.1)
With Flagellates + pathobiont	0/528	

Infections with a single species of protozoa (12.1%) (64/528) were more prevalent than co-infections (3.6%) (19/528), with statistically significant differences (p < 0.0001).

The prevalence of infection with pathogenic species (8.9%) (47/528) was similar to that of non-pathogenic species (8.7%) (46/528), and only 10 inmates (1.9%) (10/528) presented infections with both types of species.

### Distribution of intestinal parasite infections among inmates by gender, age groups and according to intestinal symptoms

The distribution of intestinal parasite infections by gender, age groups and according to intestinal symptoms is presented in Table 2. A greater number of men, 471 (89.2%), were studied compared to women, 57 (10.8%). The prevalence of infection in men 15.2% (80/528) was higher than that of women 0.6% (3/528), with statistically significant differences (p = 0.021).

**Table 2:** Socio-demographic characteristics, prevalence of intestinal parasite and bivariate and multivariate analysis of factors associated with intestinal parasites among inmates of CPP, Valencia, Spain, 2022 (* bivariate analysis; ** multivariate analysis). COR = crude Odds Ratio; AOR = adjusted Odds Ratio

Variables	Socio-demographic characteristics n/N (%)	Frequency of infection n/group (%)	Prevalence n/N (%)	COR (95% CI)	p-value*	AOR (95% CI)	p-value**
Gender							
male	471/528 (89.2)	80/471 (16.9)	80/528 (15.2)	3.68	0.021		
females	57/528 (10.8)	3/57 (5.3)	3/528 (0.6)				
Age groups (years)							
18–24	24/528 (4.5)	4/24 (16.6)	4/528 (0.8)	3.69	0.143		
25–34	115/528 (21.8)	23/115 (20.0)	23/528 (4.4)				
35–44	191/528 (36.2)	23/191 (12.0)	23/528 (4.4)				
≥ 45	198/528 (37.5)	33/198 (16.7)	33/528 (6.3)				
Intestinal symptoms							
no	468/528 (87.0)	74/468 (16.0)	74/528 (14.0)	0.78 (0.32–1.67)	0.512		
yes	60/528 (11.3)	9/60 (15.0)	9/528 (1.7)				
Education level							
Primary school or less	221/528 (41.9)	42/221 (19.0)	42/528 (7.9)	0.65 (0.39–1.08)	0.079		
Secondary school or higher	307/528 (58.1)	41/307 (13.3)	41/528 (7.8)				
Country of birth							
Spain	408/528 (77.3)	47/408 (11.5)	47/528 (8.9)	0.42 (0.23–0.78)	0.003	0.31 (0.18–0.52)	< 0.0001
Other nationalities	120/528 (22.7)	36/120 (30.0)	36/528 (6.8)				
Time spent in prison							
≤ 2 years	331/528 (62.7)	62/331 (18.7)	62/528 (11.7)	1.93 (1.11–3.45)	0.013	1.83 (1.06–3.14)	0.028
> 2 years	197/528 (37.3)	21/197 (10.6)	21/528 (4.0)				
Recent prison exit permit							
no	482/528 (91.3)	75/482 (15.6)	75/528 (14.2)	1.14 (0.44–2.61)	0.744		
yes	46/528 (8.7)	8/46 (17.4)	8/528 (1.5)				

The frequency of infection among the group of males reached 16.9% (80/471), of whom 45 (56.3%) harbored pathogenic species. In contrast, the frequency of infection among the group of females was 5.3% (3/57), two (66.7%) of them harbored pathogenic species.

Inmates were distributed in four age groups: 18–24 years (4.5%); 25–34 years (21.8%); 35–44 years (36.2%); and ≥ 45 years (37.5%) (Table 2). No statistically significant difference in intestinal parasite prevalence was detected among the age groups (p = 0.143).

The age group ≥ 45 years showed the highest prevalence of infection of 6.3% (33/528), and the age group of 18–24 years was the least infected with intestinal parasites (0.8%) (4/528). Interestingly, the frequency of infection among the inmates in both age groups was similar (16.7% and 16.6%, respectively).

All age groups showed infections with pathogenic species, but they were most common in the age group of 35–44 years, of whom 14 (60.9%) inmates harbored pathogenic species.

Intestinal symptoms (abdominal pain and/or diarrhea) were reported by 11.3% (60/528) of inmates. Although intestinal parasites are thought to cause intestinal symptoms, the prevalence among those with intestinal symptomatology was low (1.7%) (9/528) (Table 2). We did not find an association between intestinal parasite infection and intestinal symptomatology, even reporting lower prevalence of infection than those who were asymptomatic (14.0%) (74/528). The bivariate analysis showed no association (p = 0.512) between intestinal parasite infection and intestinal symptoms (COR = 0.78, 95%CI = 0.32–1.67).

In those inmates reporting intestinal symptomatology the frequency of intestinal parasite infection was 15% (9/60). Moreover, of those nine infected who reported intestinal symptomatology, five (55.6%) inmates presented pathogenic species.

### Intestinal parasite infections and potential risk factors

The socio-demographic characteristics considered as potential risk factors were education level, country of birth, time spent in prison, and a recent prison exit permit (Table 2).

Inmates with a low level of education (primary school or less) had similar prevalence of intestinal parasites as those with a higher level of education (secondary school or higher) (7.9% (42/528) and 7.8% (41/528), respectively). No association (p = 0.079) between presence of intestinal parasite and level of education was found in the bivariate analysis (COR = 0.65, 95%CI = 0.39–1.08).

Spanish inmates’ intestinal parasite prevalence was 8.9% (47/528), higher than those observed in inmates from other nationalities (6.8%) (36/528). More concretely, from the 120 foreigners, infection affected 15.8% South-Americans (19/120), 9% Europeans (11/120), 4.2% Africans (5/120) and 0.8% Asians (1/120). A statistically significant association (p = 0.003) between presence of intestinal parasite and country of birth was found in the bivariate analysis (COR = 0.42, 95% CI = 0.23–0.78).

The majority of the inmates who stayed in prison for 2 years or less had higher prevalence of infection (11.7%) (62/528) compared to those who stayed longer (4.0%) (21/528). Moreover, the bivariate analysis showed the difference in intestinal parasite infection with the time spent in prison was statistically significant (p = 0.013), and inmates who had spent 2 years or less in prison have a higher risk of intestinal parasites infection (COR = 1.93, 95%CI = 1.11–3.45).

Those who have not enjoyed a recent prison exit permit had higher prevalence of infection (14.2%) (75/528) than those who have left prison with an exit permit (1.5%) (8/528). However, the bivariate analysis showed no association (p = 0.744) between the presence of intestinal parasite and recent prison exit permits (COR = 1.14, 95%CI = 0.44–2.61).

Inmates from other nationalities and those who had spent 2 years or less in prison host more frequently intestinal parasites (30.0% and 18.7%, respectively). Multivariate analysis, to see how the significant variables acted together and simultaneously, showed that country of birth shows a negative association (less than 1) (AOR = 0.31, 95% CI = 0.18–0.52) (p < 0.0001), so the Spanish nationality could reduce the risk of infection, while less time spent in prison shows a positive association (greater than 1) (AOR = 1.83, 95%CI = 1.06–3.14), so that could favor the risk of having infection (p = 0.028).

## Discussion

Despite the fact that prison inmates are a population at higher risk than other groups of suffering from intestinal parasite infections in relation to their living conditions, information about these diseases in prison environments is still scarce. In Spanish prisons, only one study had been conducted prior to the present one in the Laboratory of Clinical Microbiology of the Hospital General Penitenciario of Madrid, reporting a total prevalence of 10.2% of intestinal parasite infections in prison population [27]. Although in our study only protozoa were detected, those authors found an elevated presence of intestinal helminths such as hookworms or *Trichuris trichiura*, probably in relation to the large proportion of African and Latin American inmates in the population of prisoners that these authors studied.

In other industrialized countries, data are only available for the 1960 and 1970 s in prisons from Canada, reaching a prevalence of 27.2% [26], and in inmates from an institution for mentally retarded patients in Finland, where the prevalence observed was 51.5% [25]. Those data are markedly higher than the ones observed in our study, but efforts aimed at improving infrastructures and sanitation systems in recent decades have surely contributed reducing the levels of parasite transmission. However, our results indicate that parasite transmission still occurs, mainly via fecal-oral and autoinfection routes.

Both the prevalences and the spectrum of parasites detected in our study were markedly different from those observed in prisons from Africa, where the prevalence ranged from 14.4% in Nigeria [19] to 72.7% in Ethiopia [13]; from Asia, where the prevalence ranged from 6.0% in inmates of the Central Jail of Kathmandu in Nepal [23] to 26.5% in Malaysia [22]; and, from South America, where the prevalence ranged from 20.2% in Midwest Brazil [29] to 80% in inmates from Rio de Janeiro (Brazil) [30]. Moreover, detection of intestinal helminths such as hookworms, *Strongyloides stercoralis, Trichuris trichiura, Ascaris lumbricoides, Hymenolepis nana, Taenia* spp., *Enterobius vermicularis* or *Schistosoma* spp. is a common feature [5, 11, 13, 15, 17, 19, 20, 22–24, 29]. In fact, Terefe et al., [18] reported a prevalence of 47.4% of intestinal helminths among inmates in the Bedele prison in Ethiopia. In contrast the absence of intestinal helminths in our study supports that the main route of parasite infection in our environment is related to direct and fecal-oral transmission and, even, water-borne infection. However, it should be noted that we have not performed either the Kato-Katz technique or the specific technique to detect *E. vermicularis* or *S. stercoralis* infection.

Despite the fact that the sizes of each population were markedly different, we observed significantly more infections in male inmates than in female inmates. This is a common feature, and, sometimes, it has been related to different hygienic practices such as washing hands after meals or after visiting the toilet [5, 13]. Probably, the greater number of inmates in the male penitentiary modules and the subsequent overcrowding can also contribute to the higher prevalence of infection [29].

We found no association between the presence of parasites and the appearance of intestinal symptoms, probably due to the similar number of pathogenic and non-pathogenic species detected. However, pathogenic species such as *E. histolytica*/*dispar* is commonly the most frequent protozoa in prisons from Africa and Latin America [5, 13, 29]. In contrast, in our study, *E. histolytica*/*dispar* was not detected since it is rarely found in Europe and just the pathogenic/potentially pathogenic species *Blastocystis* sp. (44.6%) and the pathogenic *G. intestinalis* (12.0%) showed the highest frequency among the infected inmates. We recognize that there is some controversy in relation to the role of *Blastocystis* sp. as a pathogenic entity causing human disease [22] but, under this uncertainty, we have preferred to consider it as a potentially pathogenic pathobiont.

Traditionally, a higher level of education among inmates has been associated with a lower level of intestinal parasite infections due to a greater awareness of infection risks. In fact, previous studies in Kenya and Ethiopia showed greater prevalences in unschooled inmates than in those who had been in post-primary education [5, 31]. However, we did not find differences between infected inmates with primary education (7.9%) and those with high school education (7.8%). Although it is difficult to compare, these results could be conditioned by the different levels of schooling between Spain and the countries analyzed in the previously mentioned studies.

Our results indicate that despite the significant improvement of the sanitary conditions of the prisons in Spain in the latest decades, further efforts need to be made to control fecal-oral routes of parasite transmission, either directly from person-to-person o indirectly through water or food. We also observed higher prevalence of infection in inmates with less than two years of incarceration (than in those with a longer stay in prison). On the one hand, this could be due to greater levels of immunity in long-term inmates due to continuous exposure. On the other hand, lesser time spent in prison could favor the risk of having infection. Another association found showed that the Spanish country of birth could reduce the risk of infection. With both results we can assume that foreigner’s inmates with ≤ 2 years in prison present the greatest risk of having infection. No association was found between the presence of intestinal parasite infections and recent prison exit permits.

Strikingly, in our study the prevalences of *Blastocystis* sp. and *G. intestinalis* were markedly lower than those estimated for the non-prisoner population in Spain. The prevalence of *Blastocystis* sp. was estimated at about 17% in the adult population [32] and from 3 to 7% for *G. intestinalis* in asymptomatic individuals [33]. However, data on the prevalence of intestinal parasitic infections in the general population could be highly biased and underestimated for several reasons: (i) intestinal infections with protozoa are not a compulsory notifiable disease in Spain which makes difficult to assess the burden of these infections in the country; (ii) reliable epidemiological information is restricted to certain geographical areas, whereas only incomplete or outdated information is currently available from most parts of the country; (iii) prevalences of human blastocystis and giardiasis in Spain have been mainly studied in specific populations such as children, immigrants, or hospitalized people; and (iv) a significant percentage of infections may be asymptomatic which can mask many cases. These facts make it difficult to compare the inmate and non-inmate population, so greater efforts are needed to determine the real situation of these infections inside and outside prison and to establish the necessary measures in both settings.

In summary, we have studied the current status of intestinal parasite infections in a Spanish prison. Despite the fact that it is very complex to establish a comparison with the general situation of these infections in the country, our data indicate that protozoan infections via fecal-oral transmission actively occur, which indicates the need to make efforts to improve sanitary conditions in prisons. The risk of having infection is favored in foreigners with less time spent in prison so the main recommendation would be to introduce routine parasitological tests upon entering prison and avoid, as far as possible, the transmission of intestinal parasites in those environments.

## Data Availability

The datasets analyzed during the current study are available from the corresponding author on reasonable request.

## References

[1] Feleke DG, Alemu Y, Bisetegn H, Mekonnen M, Yemanebrhane N (2021). Intestinal parasitic infections and associated factors among street dwellers and prison inmates: a systematic review and meta-analysis. PLoS ONE.

[2] Ostan I, Kilimcioglu AA, Girginkarder N, Ozyurt BC, Limoncu ME, Ok UZ (2007). Health Inequities: lower socio-economic circumstances and higher incidences of intestinal parasites. BMC Public Health.

[3] Kadio KJJO, Cissé D, Abro AL, Tounkara AF, Kéita S, Goumou S, Diaby M, Leno NN, Sylla A, Sidibé S, Camara A, Delamou A, Touré A (2021). Factors associated with intestinal parasites in the central prison of Conakry, Guinea. PAMJ - One Health.

[4] Pullan RL, Brooker SJ (2012). The global limits and population at risk of soil transmitted helminth infections in 2010. Parasit Vectors.

[5] Rop DC, Nyanchongi BO, Nyangeri J, Orucho VO (2016). Risk factors associated with intestinal parasitic infections among inmates of Kisii prison, Kissi county, Kenya. BMC Res Notes.

[6] Fitri LE, Candradikusuma D, Setia YD, Wibawa PA, Iskandar A, Winaris N, Pawestri AR (2022). Diagnostic methods of common intestinal Protozoa: current and future immunological and molecular methods. Trop Med Infect Dis.

[7] Hotez PJ, Molyneux DH, Fenwick A, Ottesen E, Enrlichsachs S, Sachs JD (2006). Incorporating a rapid-impact packaging for neglected tropical diseases with programme for HIV/AIDS, tuberculosis, and malaria. Plos Med.

[8] Wasihun AG, Teferi M, Negash L, Marugán J, Yemane D, McGuigan KG, Conroy RM, Abebe HT, Dejene TA (2020). Intestinal parasitosis, anaemia and risk factors among pre-school children in Tigray region, northern Ethiopia. BMC Infect Dis.

[9] Boga JA, Casado L, Fernández-Suarez J, Moran N, Rodríguez-Perez M, Martínez-Sela M, Pérez A, Garcia-Perez A, Menendez C, Santos S, Rodriguez-Guardado A (2020). Screening Program for Imported Diseases in immigrant women: analysis and implications from a gender-oriented perspective. Am J Trop Med Hyg.

[10] Sepahvand F, Mamaghani AJ, Ezatpour B, Badparva E, Zebardast N, Fallahi S (2022). Gastrointestinal parasites in immunocompromised patients; a comparative cross-sectional study. Acta Trop.

[11] Mardu F, Yohannes M, Tadess D (2017). Prevalence of intestinal parasites and associated risk factors among inmates of Mekelle prison, Tigrai Region, Northern Ethiopia. BMC Inf Dis.

[12] Laticevschi D. Comunicable diseases. In: Møller L, Gatherer A, Jürgens R, Stöver H, Nikogosian H, editors. Health in prisons: a WHO guide to the essentials in prison health. WHO Regional Office Europe; 2007. pp. 43–59.

[13] Mamo H (2014). Intestinal parasitic infections among prison inmates and Tobacco Farm Workers in Shewa Robit, North-Central Ethiopia. PLoS ONE.

[14] Mamman ASCR, Reuben (2014). Intestinal helminthiasis among inmates of jail Jos, Plateau State, Nigeria. W J Biol and Biol Sciences.

[15] Zida A, Sangare I, Bamba S, Sombié I, Traoré LK, Coulibaly SB, Menan H, Guiguemdé TR (2014). Intestinal parasites in prisoners in Ouagadougou (Burkina Faso). Med et Sant Trop.

[16] Okolie N (2008). Intestinal parasites distribution among inmates of Owerri prison. Int J Parasitic Dis.

[17] Ameya G, Zerdo Z, Tesfaye M, Jabesa C, Awaje A, Dejene K, Shika P, Eshetu M (2019). Intestinal parasite infections and associated factors among inmates of Arba Minch prison, southern Ethiopia: cross sectional study. BMC Infect Dis.

[18] Terefe B, Zemene E, Mohammed AE (2015). Intestinal helminth infections among inmates in Bedele prison with emphasis on soil-transmitted helminths. BMC Res Notes.

[19] Ahmed AB, Bakam H, Yayock C, Sarki M (2016). Passive surveillance of communicable diseases among inmates of Jos central prison, Nigeria. Int J Res Med Sci.

[20] Nadabo C, Ramyil MSC, Bello CSS, Ike RO, Ogundeko TO, Ihimekpen J, Suchi N, Bassi PA, Adu PJ (2020). Status of intestinal parasites in inmates of a correctional facility, Jos, Nigeria. Nigerian J Parasitol.

[21] Kamel AGM, Maning N, Arulmainathan S, Murad S, Nasuruddin A, Lai KP (1994). Cryptosporidiosis among HIV positive intravenous drug users in Malaysia. Southeast Asian J Trop Med Public Health.

[22] Angal L, Mahmud R, Samin S, Yap NJ, Ngui R, Amir A, Ithoi I, Kamarulzaman A, Lim YAL (2015). Determining intestinal parasitic infections (IPIs) in inmates from Kajang Prison, Selangor, Malaysia for improved prison management. BMC Infect Dis.

[23] Shrestha P, Shrestha D, Magar DT, Rai G, Rai KR, Rai SK (2019). Intestinal parasitic infections among prison inmates in Kathmandu Nepal. J Nepal Health Res Counc.

[24] Amit D, Kiran T, Shashwati N (2016). Prevalence of intestinal parasites and urinary pathogens among prison inmates in central jail of Bhopal (MP). Indian J Microbiol Res.

[25] Kyrönseppa H, Pettersson T (1976). The occurrence of human intestinal parasites in Finland. Scand J Infect Dis.

[26] Mecroritch E, Eaton RD (1965). Outbreak of amoebiasis among indian inmates in North Western Sackatchewan, Canada. Am J Trop Med Hyg.

[27] Alonso-Sanz M, Chaves F, Dronda F, Catalán S, González-López A (1995). Intestinal parasitoses in the prison population in the Madrid area (1991–1993). Enferm Infecc Microbiol Clin.

[28] Botero D (2012). Técnicas de laboratorio en parasitología médica. Parasitosis humanas.

[29] Curval LG, França AO, Fernandes HJ, Mendes RP, de Carvalho LR, Higa MG, de Castro Ferreire E, Cavalheiros Dorval ME (2017). Prevalence of intestinal parasites among inmates in Midwest Brazil. PLoS ONE.

[30] Figuereido-Mendes T, Fonseca EE, Guedes R, Salum N, Garrido I (1962). Treatment of penitentiary inmates for intestinal parasites in Rio de Janeiro. Am J Trop Med Hyg.

[31] Aschalew G, Belay A, Bethel N, Betrearon S, Atnad Y, Meseret A, Mengistu E, Baye G (2013). Prevalence of intestinal parasitic infections and risk factors among schoolchildren at the University of Gondar Community School, Northwest Ethiopia: a cross-sectional study. BMC Public Health.

[32] Hidalgo L, Salvador F, Sulleiro E, López I, Balladares M, García E, Paz C, Sánchez-Montalvá A, Bosch-Nicolau P, Sao-Avilés A, Molina I (2019). Evaluation of risk factors associated to detection of *Blastocystis* sp. in fecal samples in population from Barcelona, Spain: a case-control study. Eur J Clin Microbial Infect Dis.

[33] Carmena D, Cardona GA, Sánchez-Serrano LP (2012). Current situation of *Giardia* infection in Spain: implications for public health. World J Clin Infect Dis.

